# Induced pluripotent and CD34+ stem cell derived myeloid cells display differential responses to particle and dust mite exposure

**DOI:** 10.1038/s41598-023-36508-3

**Published:** 2023-06-09

**Authors:** Leonie F. H. Fransen, Martin O. Leonard

**Affiliations:** Toxicology Department, Radiation, Chemical and Environmental Hazards Directorate, UK Health Security Agency, Chilton, Harwell Campus, Didcot, OX11 0RQ UK

**Keywords:** Innate immune cells, Respiratory tract diseases

## Abstract

Myeloid cells form an essential component of initial responses to environmental hazards and toxic exposures. The ability to model these responses in vitro is central to efforts tasked with identifying hazardous materials and understanding mechanisms of injury and disease. Induced pluripotent stem cell (iPSC) derived cells have been suggested as alternatives to more established primary cell testing systems for these purposes. iPSC derived macrophage and dendritic like cells were compared to CD34+ haematopoietic stem cell derived populations using transcriptomic analysis. Using single cell sequencing-based characterisation of iPSC derived myeloid cells, we identified transitional, mature and M2 like macrophages as well as dendritic like antigen presenting cells and fibrocytes. Direct transcriptomic comparisons between iPSC and CD34+ cell derived populations revealed higher expression of myeloid differentiation genes such as MNDA, CSF1R and CSF2RB in CD34+ cells, while iPSC populations had higher fibroblastic and proliferative markers. Exposure of differentiated macrophage populations to nanoparticle alone or in combination with dust mite, resulted in differential gene expression on combination only, with responses markedly absent in iPSC compared to CD34+ derived cells. The lack of responsiveness in iPSC derived cells may be attributable to lower levels of dust mite component receptors CD14, TLR4, CLEC7A and CD36. In summary, iPSC derived myeloid cells display typical characteristics of immune cells but may lack a fully mature phenotype to adequately respond to environmental exposures.

## Introduction

Myeloid cells including macrophage (MC) and dendritic cells (DC) play an important role in innate and adaptive immune defence mechanisms in most human tissues including the lung^1,2^. These cells are necessary to maintain normal tissue homeostasis and are highly sensitive to external adverse exposures, directing appropriate immunological responses to prevent injury and disease. In addition, these cells may also be a target for exposure, producing unwanted effects and contributing to disease development^3^. Identification of adverse effects in immune cells, through toxicity testing, is a part of hazard identification efforts across many disciplines including analysis of harm from environmental exposures.

Toxicity testing efforts to date have largely relied on the use of animals for their ability to model whole systems and capture multi-cellular and multi-organ disease endpoints. Despite these advantages, species differences in responses and ethical concerns have accelerated the development and use of alternative methods of testing including in vitro systems^4,5^. This includes the field of immunotoxicology, where immunosuppression (including myelosuppression), hypersensitivity, antigenicity, autoimmunity and immunostimulatory assays have been suggested as alternatives to capture adverse immune effects^6–9^. While advances have been made in the adoption of such assays into regulatory testing systems such as the h-Clat and U-SENS skin sensitisation assays, which use the immune cell lines THP1 and U937, there still remains a lack of accepted in vitro systems capable of capturing the wide range of immune modulating effects, chemical and biological exposures can have.

One of the many roles myeloid cells, predominantly MCs, carry out is to remove particles, microbes and dead cells through a process called phagocytosis^10^. Myeloid cells can also act to phagocytose and present antigen to initiate adaptive immune responses as occurs for DCs^11^. Responses of mucosal tissues, including the lung to environmental exposures such as particles and allergens typically involves MCs and DCs as primary responders, whose responses mitigate or initiate disease processes such as those found in asthma and allergic disease^12^. Indeed, it has been demonstrated that particles including nanomaterials can have adjuvant and exacerbation effects on allergen induced disease through mechanisms involving myeloid cells^12,13^. The ability of these cell types to sense adverse effects, when applied to in vitro models of exposure has the potential to increase mechanistic understanding of adverse effects as well as to identify properties of particles and allergens of significant hazard. For example, it has been demonstrated that protease activity via protease activated receptor 2 (PAR2) activation as well as endotoxin activation of toll like receptor 4 (TLR4) mediated signalling drive immunological responses to house dust mite allergen acute exposure, independent of latter adaptive immune responses^14,15^. In order to represent in vivo settings within an in vitro model, and to reliably test for particle property effects on acute allergen exposure, one must have a fully competent model capable of appropriate molecular sensing.

CD34+ hematopoietic stem cells (HSC) isolated from human bone marrow or blood have the potential to self-renew and differentiate towards all lineages of immune cells. The ability of these cells to differentiate towards myeloid cell populations in vitro, including MC and DC using different combinations of growth and differentiation factors^16–18^ has allowed for the development of mature cell populations useful for toxicological assessments^19^. However, CD34+ derived cells are difficult to obtain in sufficient amounts from healthy individuals and have a limited lifespan limiting their applicability to in vitro toxicity assays^20^. Human monocytic cell line alternatives exist such as THP-1, however, represent relatively immature cells with abnormal phenotypes^21^, and while may be useful for specialist assays, such as skin sensitisation, are not fully suitable to capture all myeloid immunomodulatory responses primary stem cells possess.

Pluripotent stem cells such as iPSC (induced pluripotent stem cells) have been suggested as an alternative source of human haematopoietic lineage cells that may overcome many of the disadvantages associated with primary and cell line derived models^22–24^. iPSC are somatic cells reprogrammed to obtain pluripotent-like features through the introduction of specific combinations of pluripotency factors including OCT4 and SOX2^25,26^. A major advantage of the use of iPSC is an, in theory unlimited supply of donor specific cells that can be generated from easily accessible somatic cells. Furthermore, in recent years, the use of iPSC across many applications has become more accessible. Despite this, important considerations for their successful application remain, including the choice and optimisation of iPSC line and differentiation protocol^27–30^. Several studies have previously described the differentiation process of iPSC towards monocytes, MCs and DC-like cells^31–35^ including a protocol that generated monocytes with the ability to differentiate towards MCs with applications in drug screening^36,37^. In addition, CD14 sorted iPSC derived monocytes could be differentiated towards MC and DCs, functionally similar to CD34+ HSC derived cells^38^.

The use of iPSC derived myeloid cells has not been previously examined for their ability to detect hazards from particle and allergen exposure. The aim of this study was to generate different types of iPSC derived myeloid cells and compare to CD34+ HSC derived cells for their ability to respond and detect hazard from environmental triggers such as nanoparticle and dust mite exposure.

## Methods

### iPSC differentiation towards monocytes, macrophages and dendritic cells

**Figure 1:**
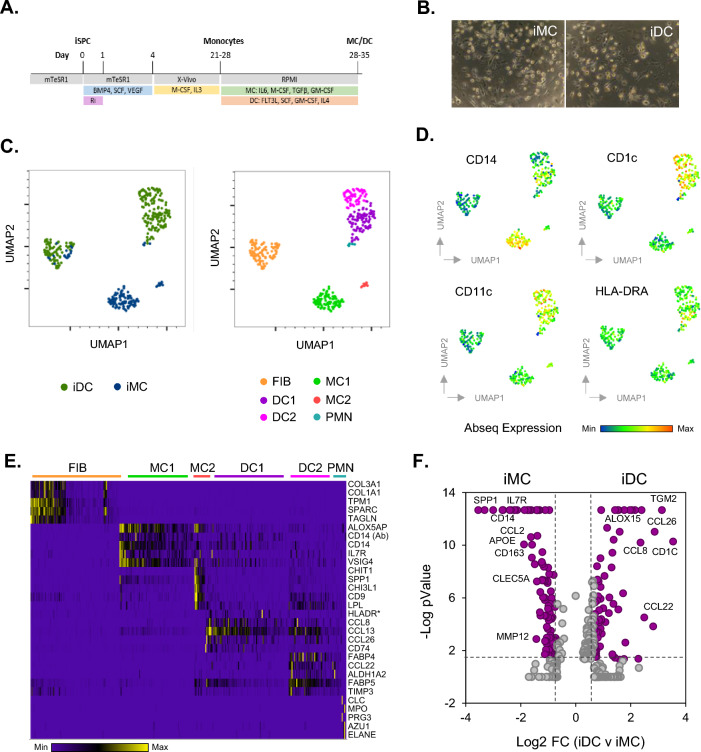
iPSC differentiation towards macrophages and dendritic cells. SBAD3 iPSC cells were differentiated towards MC cell (iMC) and DC cell (iDC) lineages over 5 weeks using differential media protocols (**A**) and visualised using phase contrast microscopy (**B**). Cells were also isolated and processed for single cell sequencing analysis (**C**–**F**). Single cell whole transcriptome gene expression profiles (**C**) as well as cell surface protein levels of lineage markers ((**D**) were visualised using UMAP allowing cell type classification. Cell type specific gene expression for DC and MC populations were also visualised as a heatmap (**E**). Statistical different gene expression between iDC and iMC populations are also displayed (**F**). *This marker is HLA-DR/CD74—AbSeq. Ab, Abseq antibody marker.

iPSC (StemBANCC; SBAD3) were obtained from NewCells Biotech (Newcastle, UK), under conditions of use outlined in the in3 MSCA-ITN project licence agreement, and differentiated towards monocytes using an established method^36^. Briefly, iPSC were cultured in 100 µL per well of a 96 well U-bottom ultra-low adherence plates at a density of 1*10^5^ cells/mL in mTeSR (StemCell Technologies) medium supplemented with 50 ng/mL BMP4, 20 ng/mL SCF, 50 ng/mL VEGF and 10 µM Rock inhibitor, in order to form embryonic bodies (EBs). 75% of this medium, without rock inhibitor supplement, was refreshed daily for 3 days. On day 5, EBs were transferred to a 6 well plate in X-VIVO 15 (Lonza) medium supplemented with 100 ng/mL M-CSF, 25 ng/mL IL3, 2 mM Glutamax, 100 U/mL Pen/Strep and 0.055 mM β-mercaptoethanol. Medium was then refreshed weekly and from week 3 onwards, monocytes (iMON) were collected from the EB cultures using a 40 µm reversible cell strainer (StemCell Technologies). Collected monocytes were further differentiated towards MC (iMC) and DC (iDC) lineages in non-tissue culture treated (NTCT) and tissue culture treated (TCT) plates respectively at a density of 100.000 cells per ml for 1 week in RPMI supplemented with low IgG FBS (10%), Glutamax (2 mM) and pen/strep. Differentiation cytokines and growth factors were also added; IL-6 (10 ng/ml), CSF1 (50 ng/ml), CSF2 (50 ng/ml) and TGFβ (2 ng/ml) for MC culture, and FL3TL (100 ng/ml), CSF1 (20 ng/ml) CSF2 (20 ng/ml) and IL-4 (20 ng/ml) for DC culture (Fig. 1A).

### CD34+ hemopoietic stem cell expansion and differentiation

**Figure 2:**
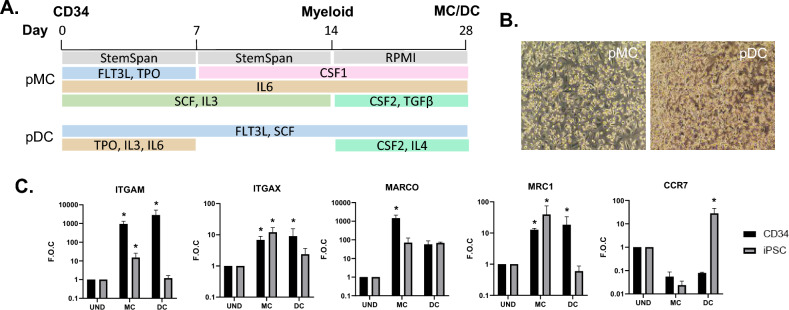
CD34+ differentiation towards macrophages and dendritic cells. CD34+ cells were differentiated towards MC cell (MC) and DC cell (DC) lineages over four weeks using differential media protocols (**A**) and visualised using phase contrast microscopy (**B**). RNA was collected from undifferentiated (UND) CD34+ cells at Day 0 and differentiated cells (MC and DC) at day 28. and examined for myeloid gene expression markers using real time qPCR (**C**). RNA from iPSC undifferentiated and differentiated iMC and IDC cultures were also collected and levels compared to CD34 derived myeloid cells (**C**). Data is visualized as fold change over undifferentiated iPSC or CD34, corrected for GAPDH, mean ± SEM (N = 3). Statistical significance compared to undifferentiated; P < 0.05 indicated as *.

Bone marrow derived CD34+ hemopoietic stem cells (StemCell Technologies, Grenoble, France) were cultured as previously described^19^. Briefly, CD34+ hemopoietic stem cells from 4 different donors were expanded for 1 week in Stemspan medium supplemented with human serum albumin (0.05%), pen/strep, FLT3L (50 ng/ml), TPO (50 ng/ml), SR-1 (1 µM), SCF (50 ng/ml), IL6 (20 ng/ml) and IL-3 (20 ng/ml) in low attachment plates. After expansion, cells were replated at a density of 5 × 10^4^ cells/ml in in either NTCT (pMC; primary MC) or TCT (pDC; primary DC) plates in RPMI supplemented with low IgG FBS (10%), Glutamax (2 mM) and pen/strep. Differentiation cytokines and growth factors were also added; IL-6 (10 ng/ml), CSF1 (50 ng/ml), CSF2 (50 ng/ml) and TGFβ (2 ng/ml) for MC culture, and FL3TL (100 ng/ml), CSF1 (20 ng/ml) CSF2 (20 ng/ml) and IL-4 (20 ng/ml) for DC culture (Fig. 2A).

### Cell treatment and cytotoxicity assessment

pMC and iMC seeded at 5 × 10^4^ cells/ml in 48 well plates were treated with cerium dioxide nanoparticles (CeO_2_ NPs; 25 µg/ml; Sigma) or silicon dioxide nanoparticles (SiO_2_ NPs; 25 µg/ml; Sigma) suspended in complete cell culture media which was sonicated (4.2*10^5^ kJ/m^3^). Cells were also treated with house dust mite soluble extracts (HDM; 15 µg/ml; Stallergenes Greer, US) alone and in combination with nanoparticles. Cell treatments were carried out for 24 h to evaluate and compare cellular responses. Dynamic Light Scattering (DLS) measurements were performed to determine the size distributions of the nanoparticle suspensions in culture medium or H2O, with or without the presence of HDM (Fig. 4A) as previously described^13^. Briefly, particles were diluted to 10^8^–10^9^ particles per mL before measurement and the Z-average size was determined using the Zetasizer Nano-ZS (Malvern) in triplicates. Cell culture media lactate dehydrogenase (LDH) concentrations were determined to analyse cellular toxicity upon exposures as previously described. LDH concentrations, indicative of necrotic cellular leakage, were determined using a commercial kit (Merck; Cat# 4744926001) according to manufacturer’s instructions.

### Real time quantitative PCR

Polymerase chain reactions (PCRs) were performed to analyse differences in gene expression between different cell types. Cells were lysed using RLT buffer (Qiagen) and homogenized using QIAShredder columns (Qiagen). mRNA was isolated using the RNeasy Mini Kit (Qiagen) and reverse transcribed to cDNA using a random hexamer-based protocol and Maxima reverse transcriptase (Thermofisher scientific) in accordance with the manufacturer’s instructions. Gene expression changes were determined by using SYBR green based real-time quantitative PCR (qRT-PCR) on the Quantstudio 6 Flex Real-Time PCR System (Applied Biosystems). Primer sequence details for each gene are detailed in supplementary Table S2) Statistical significance compared to control values was performed using one-way ANOVA and Fisher’s LSD test in GraphPad Prism software Version 8.3.0. Results are displayed as mean ± standard error of the mean (SEM).

### TempO-seq transcriptomic analysis

Targeted RNA sequencing, TempO-seq was used to characterize iPSC and CD34+ derived MCs and DCs. Cells were lysed using TempO-Seq Lysis buffer (BioClavis) and samples were outsourced and processed for quantification of 3565 gene probe sets involved in toxicological responses at BioClavis (Glasgow, UK) as previously described^39,40^. Raw TempO-Seq data was analysed externally using the TempO-SeqR software package. FASTQ files containing reads and quality scores for each sample were aligned using STAR algorithm. The obtained gene count matrix table were used for further analysis. Obtained counts for each probe-set and sample were then analysed for differential expression using DESeq2 version 1.30.0 within R^41^. Normalized counts were obtained using the DESeq2 EstimatedSizeFactors function. Endogenous control gene expression was also examined for differences between treatments and showed no significant difference (Supplementary Fig. S2).

### Single cell sequencing

Single cell suspensions of iMC and iDC cultures were collected, including adhered cells removed using 5 mM EDTA for 30 min. Single cell sequencing was carried out using the Rhapsody platform from BD Biosciences and associated reagent kits. Single cells were initially labelled with oligonucleotide tagged antibodies to distinguish sample ID and myeloid specific markers (Abseq) as detailed in supplementary Table S1. Cells from each sample were first incubated with Human BD FC block (BD) at room temperature for 10 min. Thereafter, cells were labelled with sample tags (BD Human Single-Cell Multiplexing Kit, BD) and BD AbSeq-Oligos (BD) for 45 min on ice. Cells were counted using a haemocytometer before single cell capture on microwell cartridge and lysis was performed by using the BD Rhapsody Single Cell Analysis System, according to manufacturer’s recommendations. Thereafter, Sample Tag, AbSeq and mRNA whole Transcriptome Analysis (WTA) libraries were prepared according to manufacturer’s instructions (BD). Libraries were then indexed and sequenced using Hiseq X-ten and FASTQ files were annotated using the BD WTA Multiplex Rhapsody Analysis Pipeline Version 1.8. Single cell data was further analysed using SeqGeq version 1.7.0 (FlowJo LLC, US) software. The plug-in Lex-BDSMK was used to separate out the different samples based on sample tag ID. Cell populations were identified using Seurat 3.0 plugin and visualised using UMAP dimensionality reduction based on the most highly diverse genes expressed across all cells. Differential expression between cell populations was calculated and expressed as fold change v FDR q-value significance.

## Results

### Characterisation of iPSC derived macrophage and dendritic like cells using single cell sequencing

iPSC derived MC (iMC) and DC (iDC) cells were established using sequential culture in different media components over a period of 5 weeks (Fig. 1A) revealing populations of non-adherent and adherent cells (Fig. 1B). Cells were then further characterised using single cell sequencing based analysis, where individual clusters of cells could be observed after k-means clustering analysis of whole transcriptome profiles, which for the most part did not overlap between MC and DC differentiation protocols (Fig. 1C). 6 main populations could be identified and were labelled as fibroblast like (FIB), MC like (MC1, MC2), DC like (DC1, DC2) and polymorphonuclear leukocyte like (PMN). Myeloid cell surface protein detection was also carried out and could distinguish between MC and DC populations (Fig. 1D). The monocyte and MC marker CD14 predominantly stained MC1 cells, while the DC and M2 MC markers CD1C and CD11C respectively predominantly stained the DC1 and DC2 populations. The MHCII complex marker HLA-DRA preferentially stained the DC1 population indicating a role for antigen presentation in these cells. Further characterisation of these cells is detailed in a heatmap of the top 5 most highly expressed cell type specific genes (Fig. 1E). Fibrocyte like cells were identified in both iDC and iMC populations characterised by the expression of markers involved in extracellular matrix turnover such as COL3A1 and SPARC. iDC populations displayed significantly more of these cells than the iMC population. MC1 cells stained positively for CD14 protein and mRNA along with other resting or transitional MC markers VSIG4^42^ and IL7R^43^. MC2 cells displayed specific expression of osteopontin (SPP1) and chitinase genes (CHIT1, CHI3L1), markers of more mature and adherent MC differentiation^44^. These markers are also characteristic of monocyte derived monoosteophils^32^. DC DC1 cells displayed typical antigen presentation genes HLA-DR/CD74 protein and mRNA levels as well as chemokines expressed as part of type 2 immune responses CCL8, CCL13 and CCL26^45,46^. DC2 cells also expressed the type 2 chemokine CCL22 typically expressed by M2 MCs^47^. These cells also express highly express FABP4, typically expressed by MCs of this type^48^. Differential expression between iMC and iDC populations was then calculated from the single cell transcriptomic data (Fig. 1F). This further revealed differences between differentiation protocol induction of cell type markers, including the monocyte/immature MC markers Cd163 and Ccl2, more highly expressed in the iMC population.

### iPSC and CD34+ derived macrophage and dendritic cell populations display unique and common features

**Figure 3:**
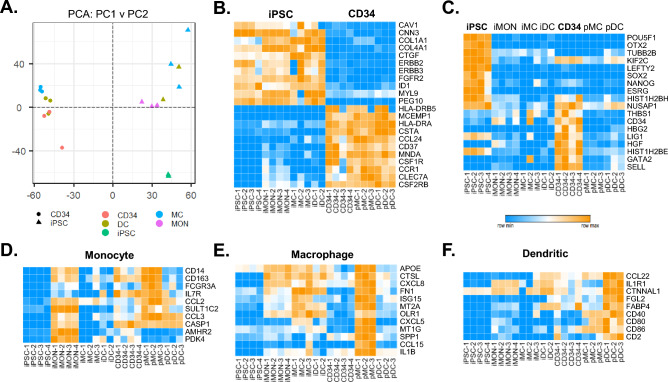
Transcriptomic comparison of iPSC and CD34+ derived myeloid cells. iPSC were differentiated towards, monocyte (iMON), MC (iMC) and DC cell (iDC) lineages while CD34+ cells were differentiated towards MC (pMC) and DC (pDC) cell lineages. Direct comparison of cell types was carried out using Tempo-seq analysis of gene expression levels (**A**–**F**). PCA analysis of gene expression differences between cell types is displayed (**A**). Genes accounting for the major differences between CD34 and iPSC PCA groupings are displayed as a heatmap of row normalised count expression (**B**). iPSC and CD34 stem cell markers are also displayed (**C**). Heatmaps of normalised count values are also displayed for selected monocyte (**D**), MC (**E**) and DC (**F**) cell markers.

We next compared iPSC derived iMC and IDC populations to primary CD34+ haematopoietic derived MC (pMC) and DC cell (pDC) populations for cell type specific marker gene expression. CD34+ cell derived pMC and pDC were derived using sequential culture of CD34+ stem cells to a range of differentiation media over a period of 4 weeks (Fig. 2A). Both adherent and non-adherent cells could be observed within both cell differentiation protocols (Fig. 2B). Cells derived from iPSC and CD34+ were then directly compared for myeloid markers using RT-PCR. Expression of ITGAM (CD11b), a typical monocyte/MC marker was highly upregulated in both MC and DC populations in CD34+ but not iPSC derived populations, when compared to undifferentiated control cells (Fig. 2C). ITGAX (CD11c) expression was found to have similar increased expression across differentiated cell types. MARCO, a cell surface scavenger receptor involved in phagocytosis of particles, bacteria and cellular debris is typically expressed on MCs^49^ and was significantly upregulated in CD34+ derived MC compared to iPSC derived DC or DC populations (Fig. 2C). MRC1 (CD206) is typically expressed on both MCs and DCs and is considered a marker of M2 differentiated MCs^50^, with functions in microbial product sensing (e.g. CpG DNA) and phagocytosis. Expression was observed as similar between iPSC and CD34+ MC populations, but only expressed in CD34+ derived DC cells (Fig. 2C). The activated DC marker CCR7 was only expressed by iPSC derived DC (Fig. 2C).iPSC and CD34+ cell populations were also directly compared using TempOseq transcriptomic analysis (Fig. 3). PCA analysis of TempOseq count data for all genes revealed distinct separation of CD34+ derived and IPSC derived cells after plotting the two largest principal components (Fig. 3A). Examination of the genes responsible revealed iPSC derived cells to have high levels of fibroblastic (e.g. COL1A1, CTGF) and proliferative cell receptors (e.g. ERBB2), not present in CD34+ derived cells. CD34+ cells alternatively displayed increased immune cell marker antigen presentation HLA markers as well as high levels of myeloid differentiation genes, including MNDA, CSFR1, CSFR2B and MCEMP1^49,51^ (Fig. 3B). We next examined markers known to identify stem cell populations of iPSC and also CD34+ Haematopoietic stem cells. Pluripotency marker genes such as POU5F1, LEFTY2 and SOX2 were uniquely expressed in undifferentiated iPSC cells, while early haematopoietic markers such as GATA2^52^ and CD34^53^ are expressed in CD34+ stem cells (Fig. 3C). Interestingly, CD34 while not expressed in undifferentiated IPSC, was expressed in iMON indicating a transition to haematopoietic progenitors during the differentiation of IPSC to MC and DC lineages. Also, of note in CD34+ cells are the expression of HBG2 and SELL, markers indicative of erythroid differentiation, which were not present in latter pMC and pDC populations. Analysis of monocyte markers including CD14, CD163 and CCL2 indicated iMON had the highest expression of the iPSC populations, while pMC had the highest expression within the CD34+ cells (Fig. 3D). MC markers representative of different subtypes^49,51^, including APOE, ISG15, SPP1 and CCL15 were most highly upregulated in pMC compared to all other cell types (Fig. 3E). There was however higher expression of the oxidised lipoprotein receptor OLR1 typically found on certain MC populations^54^ and may indicate preferential differentiation to this subtype from iPSC progenitors. DC markers such as CD40 and CD80 were preferentially upregulated in CD34+ derived pDC, with markers such as CCL22, FABP4 and IL1R1 also upregulated in pDC but also selectively increased in iDC (Fig. 3F). These latter markers may not be entirely DC specific as they are also expressed by M2 MCs^47,48^ but the expression pattern does indicate a common lineage differentiation drive in both progenitor cell populations.

### Differential response of iPSC and CD34+ derived macrophages to nanoparticle and allergen exposure

**Figure 4:**
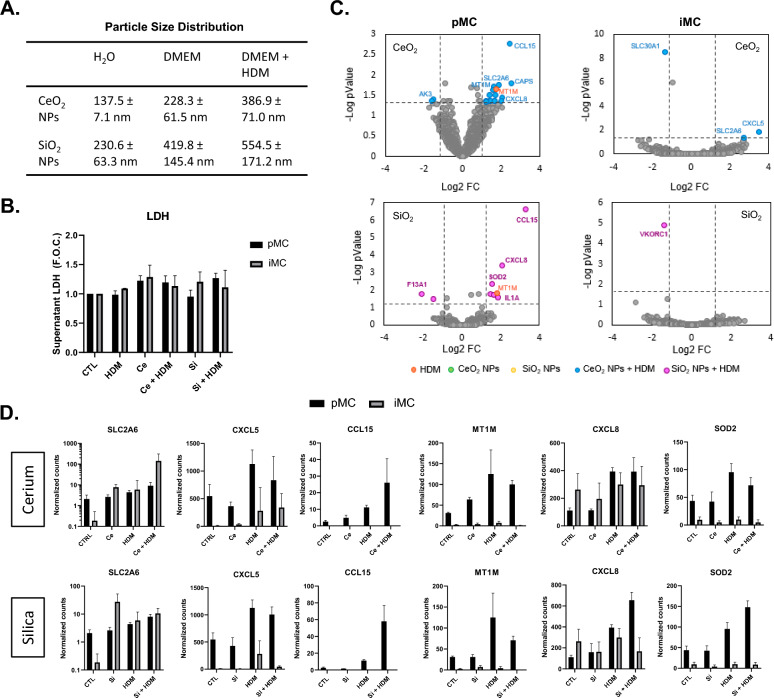
Toxicological response of iPSC and CD34+ derived myeloid cells to nanoparticle exposure. MC lineage cells were derived from iPSC (iMC) and CD34+ (pMC) and exposed to cerium dioxide (CeO2) or silicon dioxide (SiO2) (25 µg/ml) in the absence or presence of house dust mite (HDM, 15 µg/ml) for 24 h. Nanoparticle size distributions in suspension media are displayed as average +/− standard deviation (**A**). Cell culture media was analysed for levels of LDH as indicative of gross cytotoxicity (**B**). Tempo-seq analysis of gene expression levels between control and treated cells are also displayed (**C**–**D**). Volcano plots indicate differentially expressed genes between treated and control untreated cells (**C**). Differentially expressed genes from Tempo-seq analysis were examined in more detail through visualisation of normalised count data (**D**).

We next examined how differentiated MC populations responded to nanoparticle and dust mite exposure. Initial characterisation of CeO_2_ particle suspension size distributions revealed an increase in average size when suspended in cell culture media compared to distilled water (Fig. 4A). Average size distribution was also increased with addition of HDM soluble extract. Similar observations were observed for SiO_2_ particles, which did however have a larger size in all conditions when compared to CeO_2_ particles (Fig. 4A). Analysis of general cytotoxicity using LDH release revealed no significant change with any treatment (Fig. 4B). TempOseq analysis was then conducted with a summary of the main changes presented as volcano plots (Fig. 4C). Exposure to either CeO_2_ or SiO_2_ particles alone did not induce any significant changes in gene expression. House dust mite exposure altered 1 gene MT1M only in pMC with no significant change in expression observed in iMC populations (Fig. 4C). In pMC the combination of HDM with particles did however induce multiple changes in gene expression including increases in CCL15 and CXCL8, which was similar between both CeO_2_ and SiO_2_ particle co-exposures. For the most part, iMC cells did not respond to combinations of particles and HDM, except for SLC2A6 and CXCL5 induced by CeO_2_ + HDM, induction also observed in pMCs (Fig. 4C). Normalised count data for a selection of the most differentially expressed genes on TempOseq was also displayed to examine relative transcript levels across cell types and treatments (Fig. 4D). Control expression for the majority of these genes displayed a higher count level in pMC compared to iMC.

## Discussion

It was the aim of this study to generate iPSC derived myeloid cells including MC and DC lineages in order to test their toxicological responses in comparison to more established primary cell in vitro models, derived from CD34+ cells. Initial characterisation of iPSC derived cells was carried out using single cell sequencing and revealed MC and DC marker expression as expected for each differentiation protocol used. For example, CD14 mRNA and protein expression was localised to MC populations while CD1c to DC populations. Scseq analysis also revealed subpopulations of MCs and DCs within the final populations. Of note, was the identification of the DC2 population, which unlike DC1 cells did not display antigen presentation cell gene expression, such as HLA genes and CD74, typical of DCs. These DC2 cells with expression of CCL22 and FABP4 are more reliably identified as M2 like MCs, typically observed in vitro with differentiation in the presence of type 2 cytokines such as IL-4^47,55^. Indeed, the iDC protocol uses IL-4 in the final differentiation steps in our study and the identification of DC1 APC and DC M2-like cells further supports the successful implementation of these protocols to generate mature myeloid cells from IPSCs.

In addition to classical myeloid populations present on differentiation of iPSC, single cell sequencing data also identified a population of fibroblast like cells, positive for extracellular matrix proteins such as COL1A1, SPARC and ACTA2, markers not present in progenitor iPSC (Temposeq data). Similar patterns of gene expression for these fibroblastic markers were also observed in CD34+ derived populations and indicate the presence of fibrocyte or fibrocyte like cells. Fibrocytes are hematopoietic-derived cells with both mesenchymal and myeloid characteristics and have been implicated in wound repair and disease processes such as fibrosis^56^. They co-express contractile protein alpha smooth muscle actin (ACTA2), ECM components genes such as COL1A1, MC markers such as CD68 and occasionally myeloid stem cell marker CD34^56^. The presence of these cells within our differentiated IPSC and CD34+ populations further indicate the protocols used are capable of producing a broad range of myeloid cell types. Direct comparison of fibrocyte marker levels do reveal some differences between iPSC and CD34 derived populations with IPSC displaying higher levels of ECM collagens. This difference has been observed previously where CD14 + purified iPSC cell derived MCs and DC protocols produced higher levels of fibrocyte and ECM collagen markers COL1A2 and COL3A1, when compared to MCs and DCs derived from primary blood derived monocytes^38^. Further similarities in this study to our own were also observed with increased activated DC marker CCR7 and decreased HLA genes observed in iPSC derived when compared to primary cell derived lineages^38^.

Despite successful differentiation of iPSC cells to different myeloid lineages, it is clear that significant differences still exist between these and CD34+ derived cells, as can observed on direct comparison of TempOseq gene expression data. Specifically, if one compares markers common to all CD34 populations, absent or largely reduced in iPSC derived populations, it is clear CD34+ cells appear to have particular immune cell functions and marker expression indicative of a more mature and specialised nature even within the originating CD34+ stem cell population. For example, HLA genes, critical for antigen presentation functions in DC and B-cells among others^57^ are much more strongly expressed in CD34, than iPSC cells. Similarly, genes found in monocytes (MNDA, CSF1R and CCR1)^58,59^, mast cells (MCEMP1)^57,60^, B-cells (CD37)^61^ and M2 MCs (CCL24)^47,55^ were all present in CD34+ populations while absent in iPSC derived. These differences indicate further diversity within the CD34+ derived cells, but more importantly indicate a competency for certain myeloid functions and responses in these cells that may not be captured from iPSC derived. Therefore, comparison between cell source lineages for toxicological responses must take into consideration the difference in cell type differentiation.

With this in mind, we compared both iPSC and CD34+ derived MCs for their response to nanoparticle + /− house dust mite exposure after 24 h of exposure. No significant differences in gross cytotoxicity were observed between any treatment indicating any changes in gene expression were independent of cell death pathway activation. There were however significant changes in gene expression, which overall were more pronounced for CD34+ derived cells than iPSC derived. Particles alone did not have any significant effect on gene expression, indicating that these cells are incapable of responding to particles, or that these particles at this dose have limited toxicological impact. There was however significant difference in MT1M expression with HDM alone. Metallothionein expressing MCs have been identified within the lung and have been suggested to play a role in tissue defence, repair and homeostasis^62,63^. While limited effects were observed with each treatment alone, a combination of particle and dust mite produced a significant number of differentially expressed immune regulatory genes in CD34+ derived cells, which was not observed in iPSC derived cells. As we have previously demonstrated nanoparticle mediated exacerbation of dust mite induced type 2 inflammation in mice^64^, it is possible that such effects may be mediated through a mechanism involving combinatorial effects on myeloid MCs present within our CD34+ population. As such effects were not observed to the same extent in iPSC derived cells, it may call into question, their applicability to detect this type of hazard in vitro.

The ability to interrogate transcriptomics data between cell types may provide some information as to why at a molecular level, iPSC derived MCs are less sensitive than CD34+ for detecting combined particle/dust mite effects. One of the main drivers of acute responses to dust mite exposures, is not the allergen proteins themselves, but associated endotoxin levels^65^. Endotoxins including LPS activate toll like receptor (TLR) signalling through binding TLR4 and the co-receptor CD14^66^ to initiate inflammatory signalling in mainly monocytes and MCs. As the levels of CD14 and TLR4 were higher in pMC compared to iMC populations, it is suggested that the lack of this receptor complex in iMC may account for the lack of responses observed in this iPSC derived population. Interestingly the iMON population had higher levels of CD14 than iMC and may contain more responsive cell types to endotoxin and dust mite exposures. This observation also demonstrates the complexity of transition between differentiation states, as both iPSC and CD34 cells had the same media and conditions for the last week of their differentiation protocols. The origin and differentiation state of the precursor cell state are likely to be central to final differentiation. It is also recognised that the lack of responsiveness of iMC could be due to lower expression of other molecular receptors and phagocytotic mechanisms such as dectin-1 (CLEC7A), which has been demonstrated as mediating inflammatory effects in response to HDM^67^ and was significantly lower in iMC compared to pMC cells. Similar observations could be made for other molecules such as CD36, demonstrated to be involved in HDM induced allergy development^68^.

Despite the overall lack of responsiveness of iPSC derived myeloid cells, there were two genes induced upon the combination of CeO_2_ particles/HDM, CXCL5 and SLC2A6 in iMC. CXCL5 is a chemokine induced by inflammatory stimuli from immune cells such as MCs^69^. Similarly, the monosaccharide transporter SLC2A6, which is involved in glycolysis regulation in inflammatory MCs^70^, was upregulated by combined exposure, an alteration which was also observed in CD34+ derived pMCs. These results indicate that while the sensitivity to detect this set of exposures is diminished in iMC, there may still be some level of detection present, perhaps reflective of a smaller number of sensitive cells within the overall population. However, not all sensitivities are present within the iMC. When we examine the most highly regulated gene CCL15, by both particle type combined treatments in pMC, expression was absent from iMC treatments. This chemokine is a potent chemoattractant for monocytes^71^. Its expression is also inducible in monocytes and monocytoid cells through activation of NF-κB signalling^72^. More recently it has been demonstrated as derived from eosinophils and to promote type 2 airway inflammation^73^. It is therefore unlikely that this model of iPSC derived MC are capable of detecting mechanisms of inflammation involving this chemokine.

It is also important to outline the limitations of this study, such that a more complete picture of how these findings should be considered in the broader context. The first limitation is that only one iPSC line was used. It has long been acknowledged that different iPSC cell lines have different capacities to differentiate towards certain germ layers and lineages^30^, which has been mainly attributed to differences in epigenetic patterning and genetic background^30,74,75^. While it is clear the SBAD3 line used in our study was capable of haematopoietic progenitor differentiation and to display markers of myeloid lineages, the possibility exists that a different iPSC line may be better capable of differentiation to more mature phenotypes as observed using CD34+ primary stem cells. Another limitation of this study is that only one type of differentiation protocol was used. It is therefore also possible that modification of this protocol, or the use or optimisation of a fundamentally different protocol^76^ may result in further improvements in differentiation towards primary myeloid cell like phenotype. Subsequently, this may also improve the capacity to detect environmental exposure adverse effects within in vitro toxicity testing endeavours. Moreover, it is possible that generation of iPSC lines using different pluripotency protocols^26^ may also improve downstream hematopoietic differentiation and further optimise towards a more mature phenotype.

In summary IPSC derived myeloid cells using classically defined MC and DC protocols produced a broad range of cell types of myeloid origin. Differences in the relative abundance of cell populations are present, that likely underpin the difference in response to particle and dust mite stimulation. These differences may involve expression of CD14, TLR4 and other phagocytic receptors within the pMC population compared to iMC.

## Supplementary Information

Supplementary Information.

## Data Availability

The datasets used and/or analysed during the current study are available from the corresponding author on reasonable request.

## Abbreviations

iPSC: induced pluripotent stem cells
iMC: iPSC derived macrophage cells
iDC: iPSC derived dendritic cells
Fib: Fibrocyte
PAR2: Protease activated receptor 2
TLR4: Toll like receptor 4
CTRL: Control
DC: Dendritic cell
pDC: Primary CD34+ derived dendritic cells
FC: Fold change
F.O.C: Fold over control
HDM: House dust mite
MC: Macrophage cell
pMC: Primary CD34+ derived macrophage cells
LDH: Lactate dehydrogenase
SEM: Standard error of the mean
