# Supplementary material for: Induced pluripotent and CD34+ stem cell derived myeloid cells display differential responses to particle and dust mite exposure
Source: Sci Rep. 2023 Jun 9;13:9375. doi: 10.1038/s41598-023-36508-3 (PMC10256772; doi:10.1038/s41598-023-36508-3)
Supplement: Supplementary file 1 — Supplementary Information. [file 41598_2023_36508_MOESM1_ESM.pptx]

## Slide 1
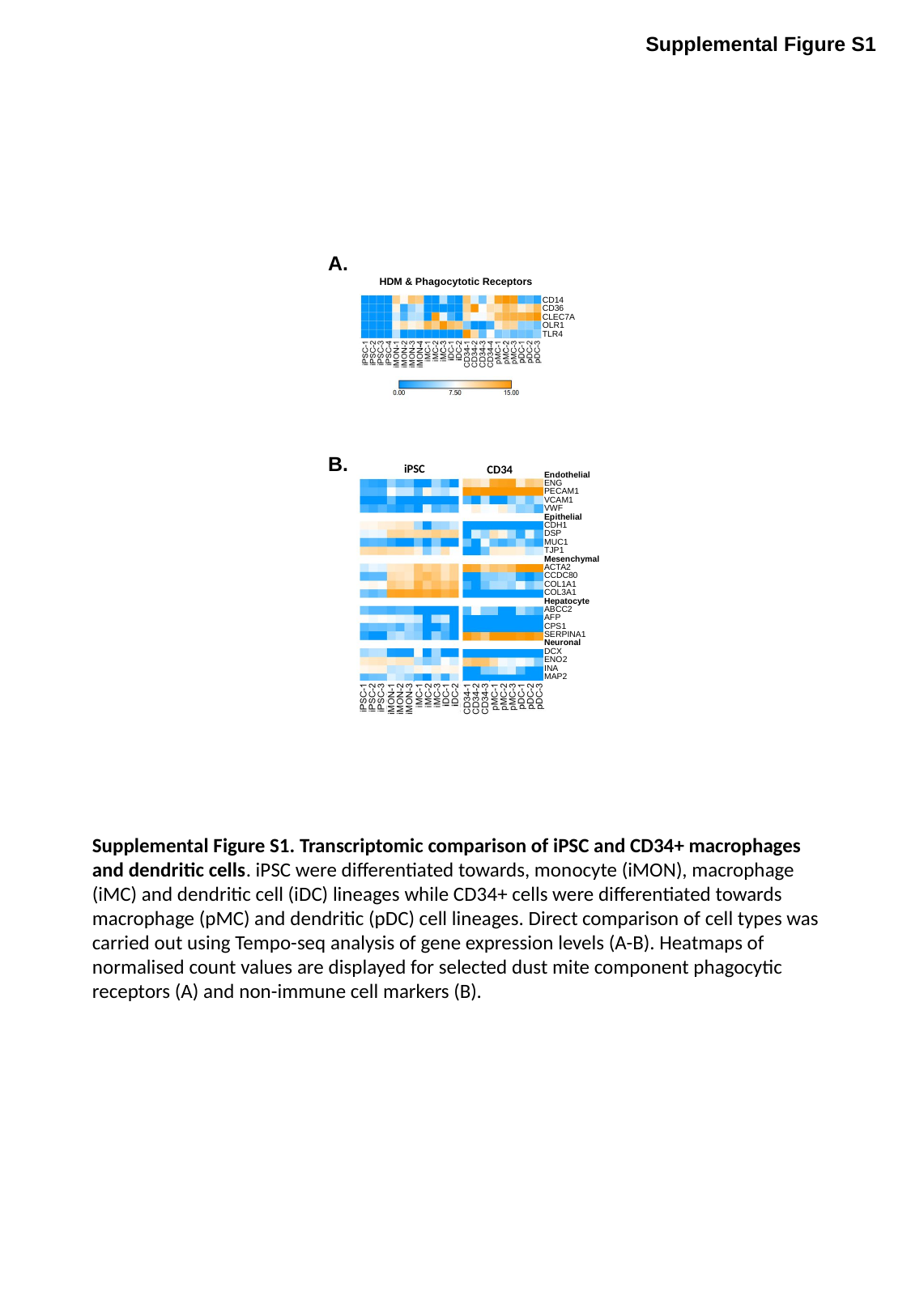

Supplemental Figure S1
A.
HDM & Phagocytotic Receptors
CD14
CD36
CLEC7A
OLR1
TLR4
B.
iPSC
CD34
Endothelial
ENG
PECAM1
VCAM1
VWF
Epithelial
CDH1
DSP
MUC1
TJP1
Mesenchymal
ACTA2
CCDC80
COL1A1
COL3A1
Hepatocyte
ABCC2
AFP
CPS1
SERPINA1
Neuronal
DCX
ENO2
INA
MAP2
Supplemental Figure S1. Transcriptomic comparison of iPSC and CD34+ macrophages and dendritic cells. iPSC were differentiated towards, monocyte (iMON), macrophage (iMC) and dendritic cell (iDC) lineages while CD34+ cells were differentiated towards macrophage (pMC) and dendritic (pDC) cell lineages. Direct comparison of cell types was carried out using Tempo-seq analysis of gene expression levels (A-B). Heatmaps of normalised count values are displayed for selected dust mite component phagocytic receptors (A) and non-immune cell markers (B).

## Slide 2
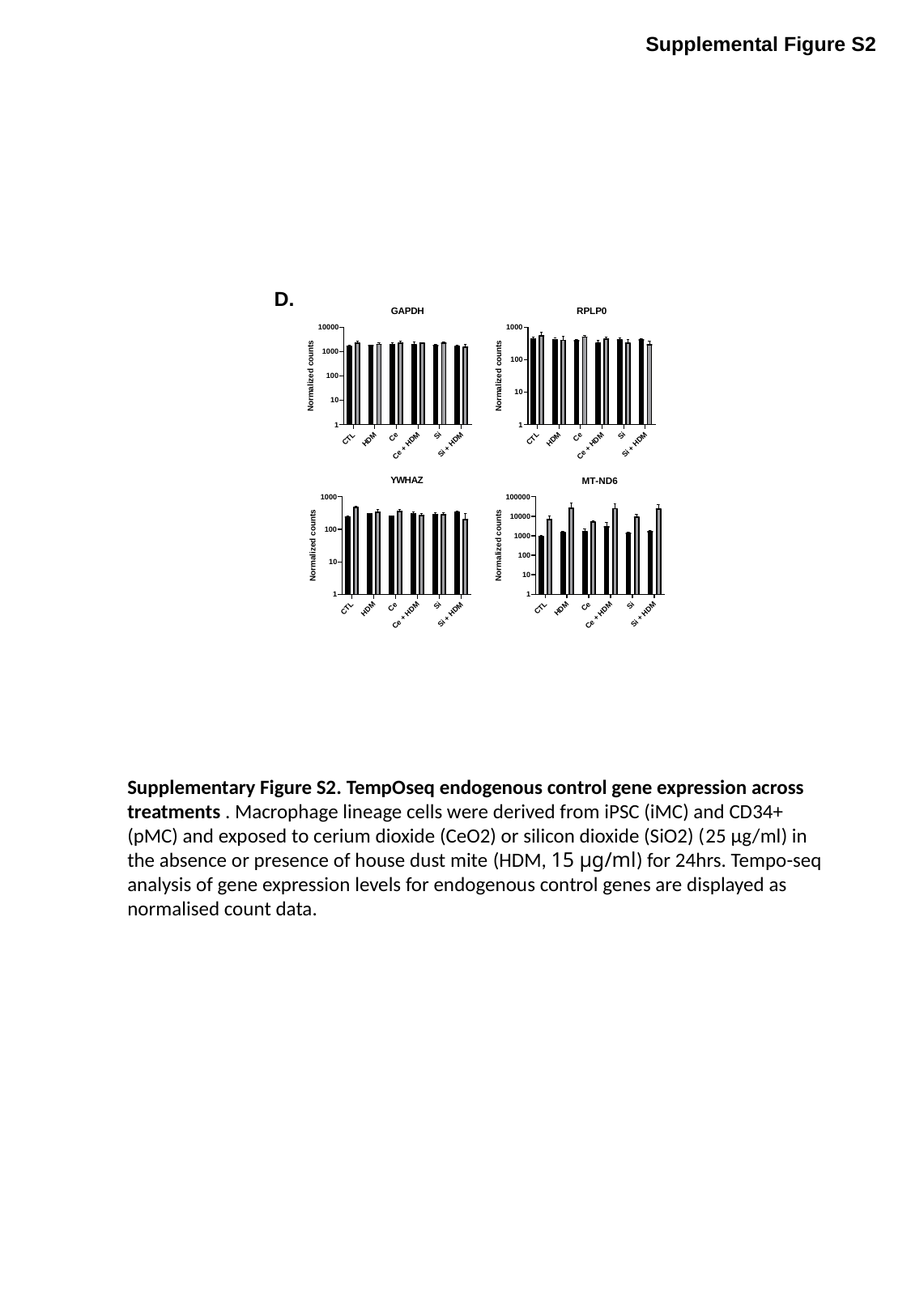

Supplemental Figure S2
D.
Supplementary Figure S2. TempOseq endogenous control gene expression across treatments . Macrophage lineage cells were derived from iPSC (iMC) and CD34+ (pMC) and exposed to cerium dioxide (CeO2) or silicon dioxide (SiO2) (25 µg/ml) in the absence or presence of house dust mite (HDM, 15 µg/ml) for 24hrs. Tempo-seq analysis of gene expression levels for endogenous control genes are displayed as normalised count data.

## Slide 3
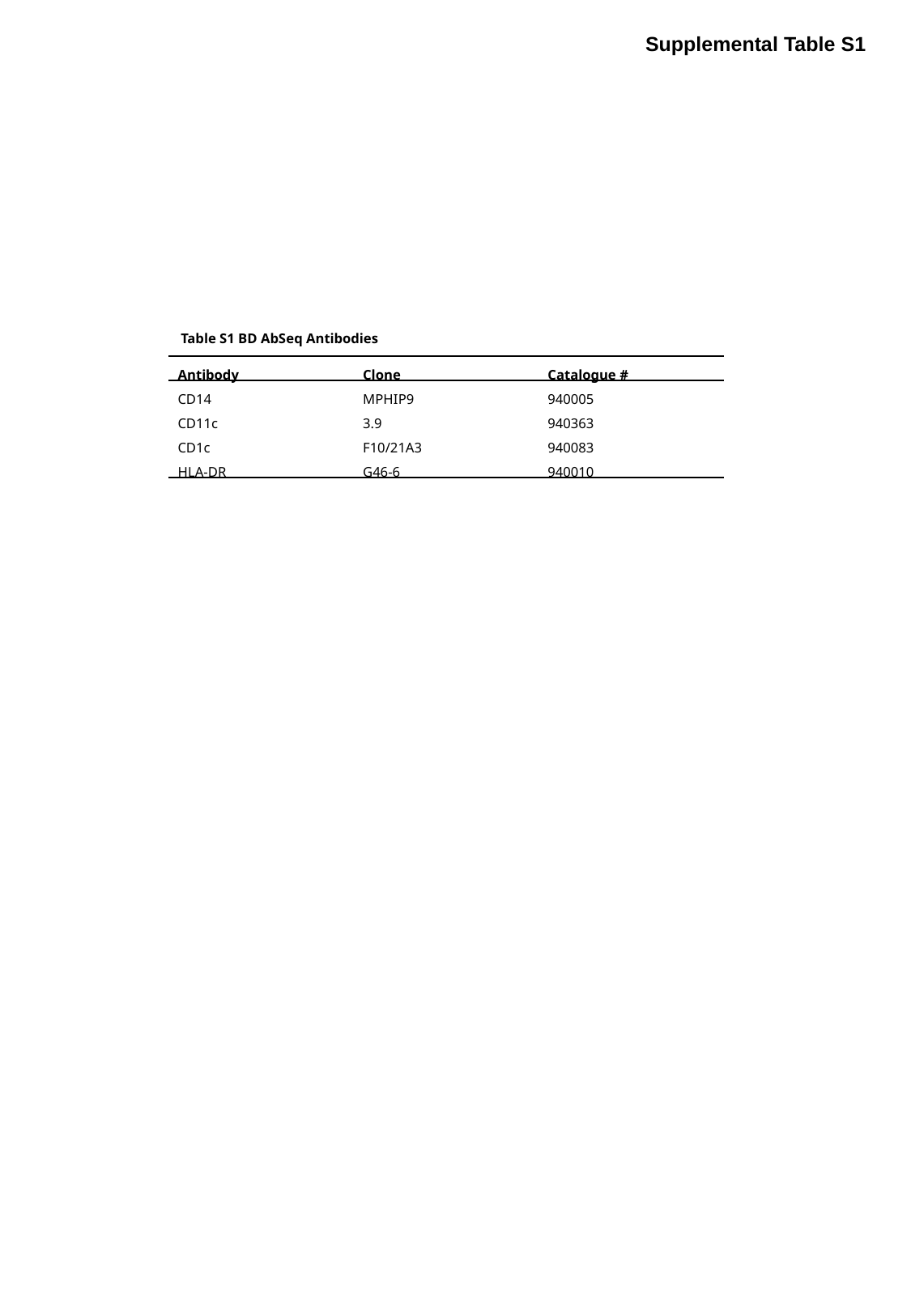

Supplemental Table S1
Table S1 BD AbSeq Antibodies
| Antibody | Clone | Catalogue # |
| --- | --- | --- |
| CD14 | MPHIP9 | 940005 |
| CD11c | 3.9 | 940363 |
| CD1c | F10/21A3 | 940083 |
| HLA-DR | G46-6 | 940010 |

## Slide 4
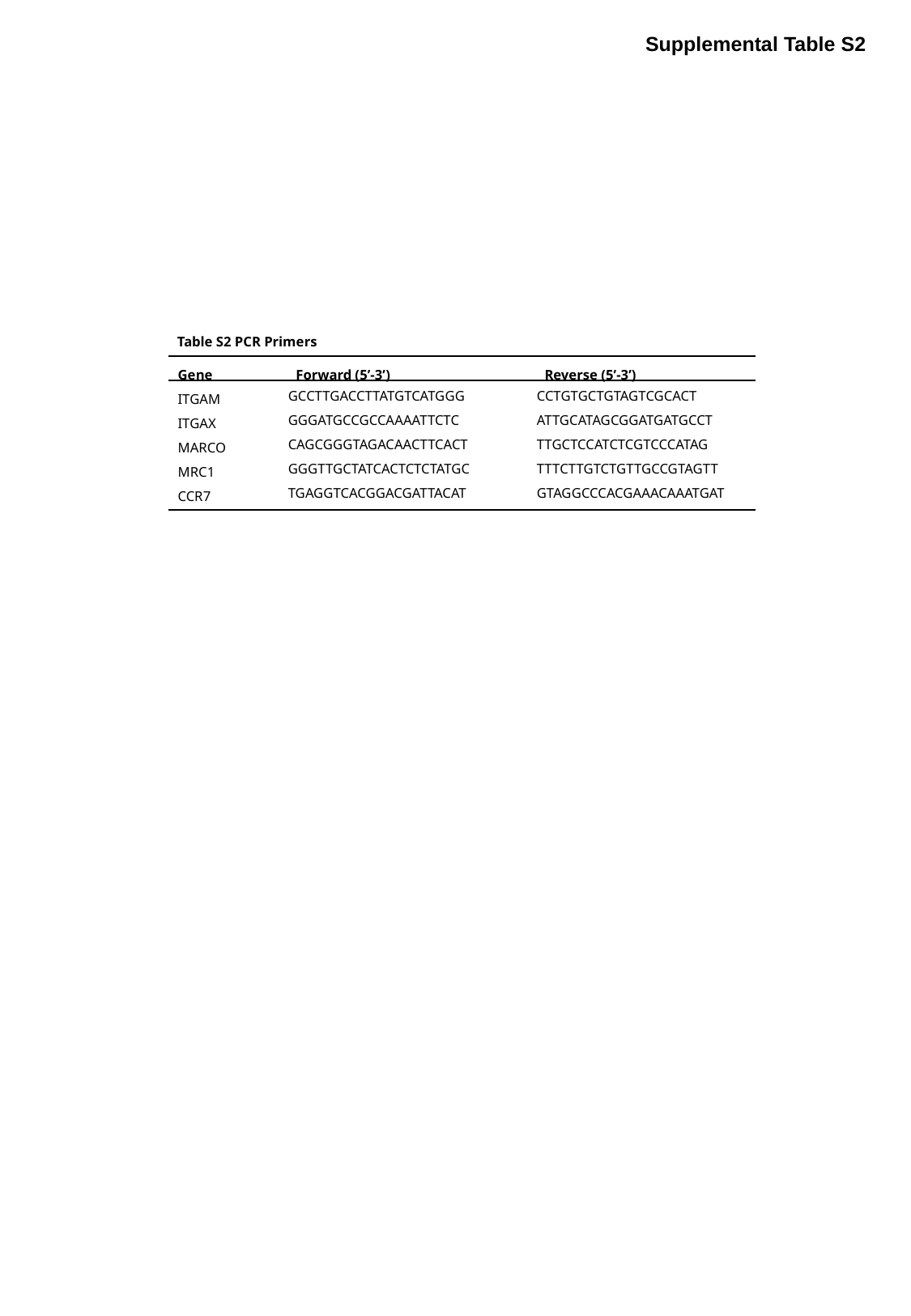

Supplemental Table S2
Table S2 PCR Primers
| Gene | Forward (5’-3’) | Reverse (5’-3’) |
| --- | --- | --- |
| ITGAM | GCCTTGACCTTATGTCATGGG | CCTGTGCTGTAGTCGCACT |
| ITGAX | GGGATGCCGCCAAAATTCTC | ATTGCATAGCGGATGATGCCT |
| MARCO | CAGCGGGTAGACAACTTCACT | TTGCTCCATCTCGTCCCATAG |
| MRC1 | GGGTTGCTATCACTCTCTATGC | TTTCTTGTCTGTTGCCGTAGTT |
| CCR7 | TGAGGTCACGGACGATTACAT | GTAGGCCCACGAAACAAATGAT |
| | | |
